# Autologous tissue reconstruction sternal cleft: a rare congenital malformation

**DOI:** 10.1093/jscr/rjab550

**Published:** 2022-03-04

**Authors:** Atthawit Mongkornwong, Laliphat Kongpanichakul, Niti Tawaranurak, Orawan Chansanti, Voravit Chitithavorn, Apirag Chuangsuwanich

**Affiliations:** Division of Plastic and Reconstructive Surgery, Department of Surgery, Faculty of Medicine Songklanagarind Hospital, Prince of Songkla University, Songkhla, Thailand; Division of Plastic and Reconstructive Surgery, Department of Surgery, Faculty of Medicine Songklanagarind Hospital, Prince of Songkla University, Songkhla, Thailand; Division of Plastic and Reconstructive Surgery, Department of Surgery, Faculty of Medicine Songklanagarind Hospital, Prince of Songkla University, Songkhla, Thailand; Division of Plastic and Reconstructive Surgery, Department of Surgery, Faculty of Medicine Songklanagarind Hospital, Prince of Songkla University, Songkhla, Thailand; Division of Cardiovascular Thoracic Surgery, Department of Surgery, Faculty of Medicine Songklanagarind Hospital, Prince of Songkla University, Songkhla, Thailand; Division of Plastic and Reconstructive Surgery, Department of Surgery, Faculty of Medicine Siriraj Hospital, Mahidol University, Bangkok, Thailand

## Abstract

Sternal clefts are rare congenital chest-wall deformities, which can be complete or incomplete; therefore, reconstruction with autologous tissue is essential to protect the heart and prevent respiratory infection. In this report, we present the case of a 16-month-old baby girl from a preterm, twin pregnancy with a partial superior sternal cleft. A moist dressing for promoting wound healing was used until cutaneous layer complete epithelialization at which time we performed reconstruction with autologous tissue. After surgery the patient recovered with close-to-natural chest wall contour and adequate heart and lung function.

## INTRODUCTION

A sternal cleft is a rare congenital disease, with a reported incidence of 1:100 000 cases per live birth and <1% of chest wall congenital defects [1]. The development of the sternum starts during the sixth week of gestational age [2]. Sternal clefts can be either complete or incomplete, with an incomplete superior sternal cleft in either a V or U shape. Most patients are most prone to developing respiratory infections due to paradoxical respiratory movements in the defect area. Inferior partial sternal clefts are rare and usually associated with thoracoabdominal ectopia cordis [3].

An early diagnosis, such as during the intrauterine stage with ultrasound and early correction, provides benefits for the thoracic cage’s elasticity and a cosmetic outcome [4]. The surgery aims to protect the heart from direct injury and respiratory tract infection, and ensure a good cosmetic outcome [1]. Varying surgical techniques for correcting a partial sternal cleft, such as using periosteum with a chondral graft [5], iliac bone grafts [6], wedge excision of the inferior part [7], alloplastic reconstruction [1] and muscle flap [8] have been reported in the literature [9].

**Figure 1:**
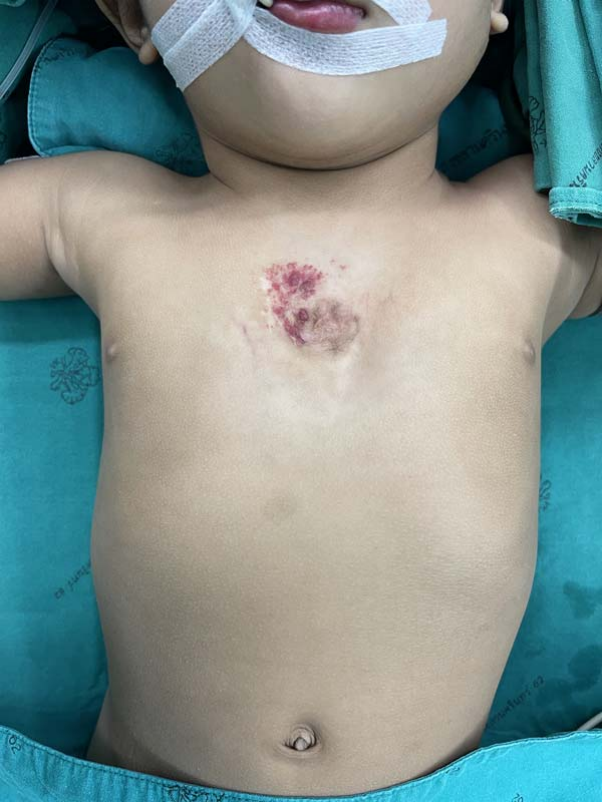
Preoperative picture.

**Figure 2:**
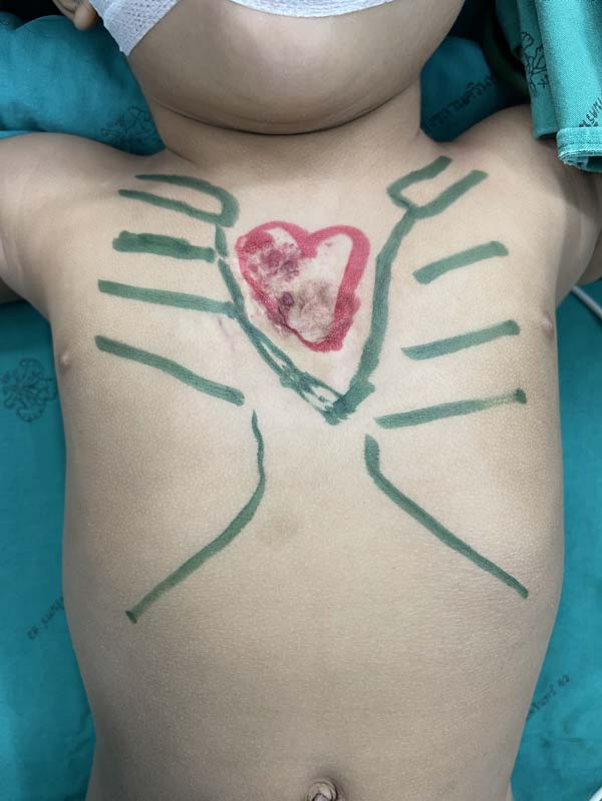
Preoperative marking.

**Figure 3:**
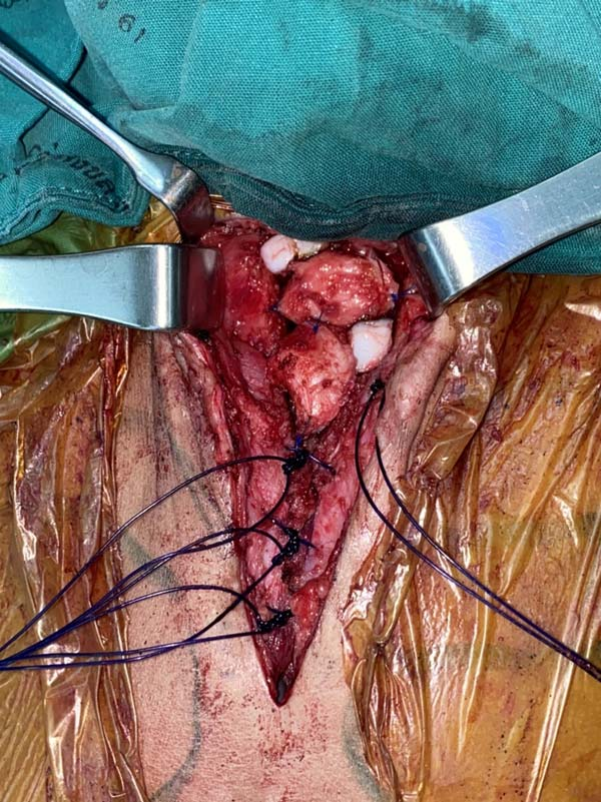
Autologous cartilage graft.

**Figure 4:**
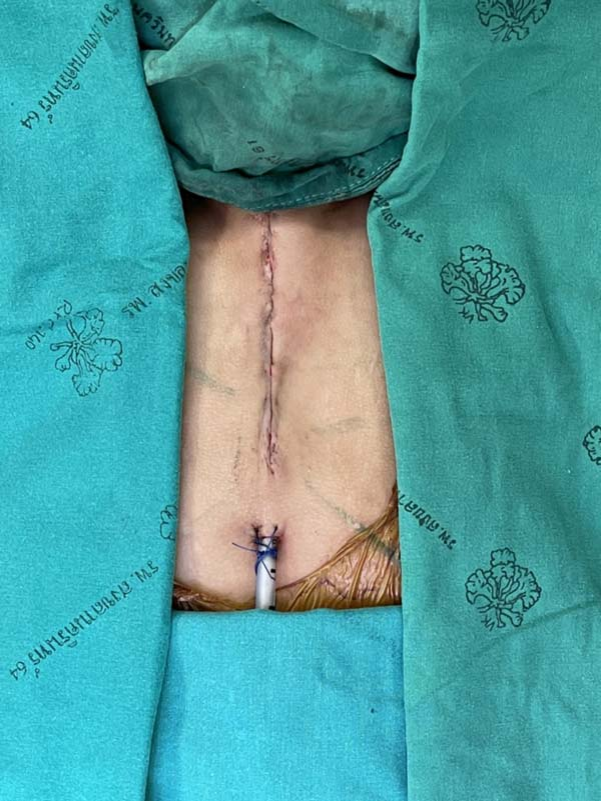
Skin closure.

## CASE REPORT

A 16-month-old baby girl with a birth weight of 1060 g and uneventful antenatal care, was referred from rural hospital to our institution. Our initial examination showed cyanosis with a sternal defect with only a thin cutaneous covering over the heart. Her history was significant for mild hypoplastic arch, with aberrant RSCA from CTA and ECC with a mean normal, her condition could be controlled with antihypertensive drugs. She had other malformations, notably scoliosis without surgical correction and parotid hemangioma, which was treated with propranolol. After managing all associated anomalies, a CT scan revealed a partial superior sternal cleft with a thin skin covering the heart; all other physical examinations were normal. First, we placed a dressing with a moist gel until cutaneous layer complete epithelialization, which we maintained for 6 months with regular dressing changes. When she was deemed ready for the next step, operative reconstruction of the defect was then performed. First, the skin was marked at the costal margin (Figs 1 and 2), then skin infiltration with diluted 1% xylocaine with adrenaline 1:20 000 1 ml in normal saline 20 ml was administered. We waited for 7 min for the adrenaline to take effect, and then meticulously dissected the cutaneous layer until reaching the costal margin on both sides. After exposing the total costal margin and xiphoid process (upper: sternal notch, lower: xyphoid process and lateral: costal margin), we used scissors and a scalpel to excise the lower part of the sternum at the V shape. Then we used autologous cartilage from the xiphoid process to bridge the defect over the heart and secured this bilaterally with polypropylene 2-O non-absorbable sutures to the cleft edge (Fig. 3). Follow the closure of the defect, the hemodynamics were observed for intra-thoracic pressure and cardiac function for 5 min. The midline was covered with cutaneous tissue and secured with vicryl 5–0 and polypropylene 6–0 (Fig. 4).

**Figure 5:**
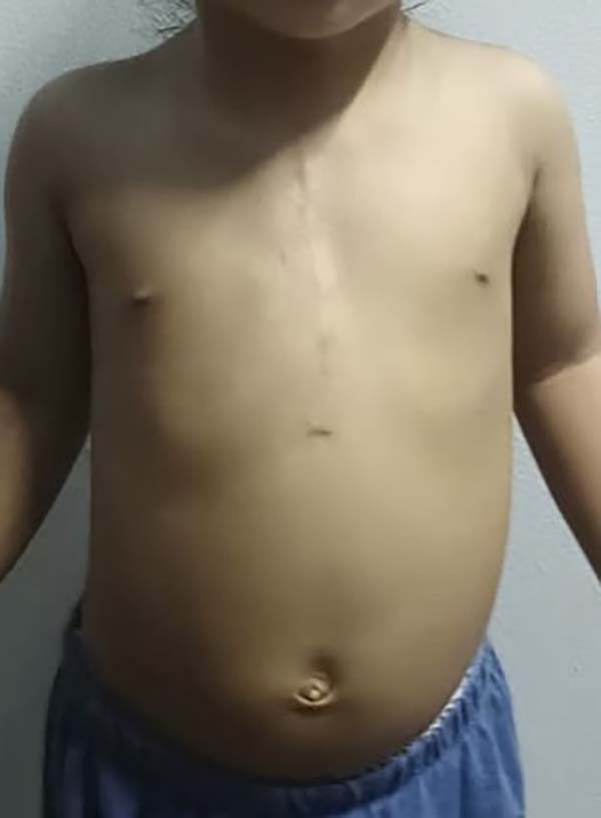
Postoperative follow up 6 months.

The patient had an uneventful postoperative course and was discharged home on the fifth day postoperatively. The child was doing well and had an excellent cosmetic result with no complications at the 3-month follow-up (Fig. 5).

## DISCUSSION

Sternal clefts are a rare congenital defect that involve the anterior chest wall, with an incidence of <1% of all chest wall congenital deformities [4]. Early diagnosis by ultrasound is easily made and helpful in evaluating associated anomalies, especially cardiac defects. Upon discovering this patient had a sternal cleft, a multidisciplinary team was consulted and associated malformations were excluded. Radiology investigation involving a CT scan with 3D reconstruction and a magnetic resonance image of the chest wall were obtained. Other investigations, such as echocardiogram and abdominal ultrasound were also conducted. Surgical repair was undertaken to protect the heart from direct injury and respiratory tract infection, with care taken for a good cosmetic outcome. In case of sternal cleft early repair within the first 3 months preferred due to the elasticity of the thoracic wall and for better cosmetic results. Autologous tissue or alloplastic can be used for the repair, depending on the case.

Reconstruction of a sternal cleft is a challenging situation, as many complications can occur including pneumothorax, massive blood loss, cardiac injury and arrhythmia. Therefore, advanced monitoring and advanced anesthetist are essential. Increasing the thickness of the skin prior surgery is essential so we used a moist dressing to allow a healing process until the skin reached adequate thickness. And autologous cartilages from the resected inferior part of the sternum were used to obliterate space at superior part of sternal defect as it can decrease tension between the costal ridge during closure. In addition, autologous cartilage has better wound healing properties than others. A near-perfect reconstruction result can be achieved by careful preoperative planning with a multidisciplinary team, advanced monitoring and proper anesthesia surgical techniques.

## CONCLUSION

The sternal cleft is a rare congenital disease. The management began during intrauterine diagnosis followed by post-delivery planning with a multidisciplinary team to screen for associated anomalies, and surgical planning. Early treatment of other malformations was done before surgical correction of the sternal cleft including a facial hemangioma. Sternal cleft repairs should be done earlier, and autologous tissue reconstruction could be suitable and safe for sternal cleft repair.
